# Repairable *ex vivo* model of functional and degenerative mitral regurgitation

**DOI:** 10.1093/ejcts/ezad371

**Published:** 2023-11-10

**Authors:** Hayato Morimura, Yusei Okamoto, Jumpei Takada, Minoru Tabata, Kiyotaka Iwasaki

**Affiliations:** Cooperative Major in Advanced Biomedical Sciences, Joint Graduate School of Tokyo Women's Medical University and Waseda University, Waseda University, Tokyo, Japan; Department of Modern Mechanical Engineering, Graduate School of Creative Science and Engineering, Waseda University, Tokyo, Japan; Department of Integrative Bioscience and Biomedical Engineering, Graduate School of Advanced Science and Engineering, Waseda University, Tokyo, Japan; Department of Cardiovascular Surgery, Juntendo University, Tokyo, Japan; Cooperative Major in Advanced Biomedical Sciences, Joint Graduate School of Tokyo Women's Medical University and Waseda University, Waseda University, Tokyo, Japan; Department of Modern Mechanical Engineering, Graduate School of Creative Science and Engineering, Waseda University, Tokyo, Japan; Department of Integrative Bioscience and Biomedical Engineering, Graduate School of Advanced Science and Engineering, Waseda University, Tokyo, Japan; Institute for Medical Regulatory Science, Waseda University, Tokyo, Japan

**Keywords:** Mitral valve, Mitral regurgitation, Mitral valve repair, Preclinical, *Ex vivo*, Model

## Abstract

**OBJECTIVES:**

Transcatheter mitral valve repair is an emerging alternative to the surgical repair. This technology requires preclinical studies to assess efficacy in mitigating mitral regurgitation (MR). However, *ex vivo* MR models are not established. We developed 2 novel repairable models, functional and degenerative, which can quantitatively assess regurgitation and effect of intervention.

**METHODS:**

We used porcine mitral valves and a pulsatile flow circulation system. In the functional MR model, the annulus was immersed in 0.1% collagenase solution and dilated using 3D-printed dilators. To control the regurgitation grade, the sizes of the dilator and silicone sheet in which the valve was sutured to were adjusted. Chordae of P2 were severed in the degenerative model, and the number of severed chordae was adjusted to control the regurgitation grade. Models were repaired using the edge-to-edge or artificial chordae technique.

**RESULTS:**

The mean regurgitant fraction of the moderate–severe functional and degenerative models were 47.9% [standard deviation (SD): 2.2%] and 58.5% (SD: 8.0%), which were significantly reduced to 28.7% (SD: 4.4%) (*P* < 0.001) and 26.0% (SD: 4.4%) (*P* < 0.001) after the valve repair procedures. Severe functional model had a mean regurgitant fraction of 59.4% (SD: 6.0%).

**CONCLUSIONS:**

Both functional and degenerative models could produce sufficient MR levels that meet the interventional indication criteria. The repairable models are valuable in evaluating the efficacy of valve repair procedures and devices. The ability to control the amount of regurgitation enhances the versatility and reliability of these models. These reproducible models could expedite the development of novel devices.

**Figure:**
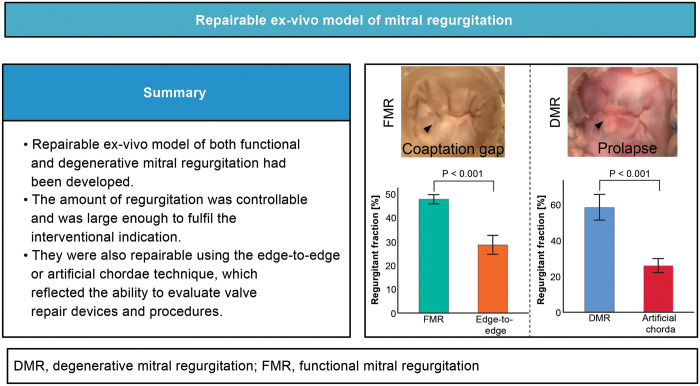


## INTRODUCTION

Mitral regurgitation (MR) affects 24.2 million people around the world [1]. However, only 15% of these patients with MR actually undergo surgical treatment [2]. The high operative risks may be due to advanced age, heart failure and other comorbidities [3]. To fulfil the unmet needs of these inoperable patients, minimally invasive treatments, such as transcatheter mitral valve repair (TMVr), have emerged. Edge-to-edge TMVr using MitraClip (Abbott Vascular, Menlo Park, CA, USA) had affected MR treatment. However, issues such as postprocedural mitral stenosis and lower procedural success in the non-A2P2 location of MR remain [4, 5]. To overcome these issues and benefit more patients, our ultimate goal is to develop a novel TMVr device. Mitral valve repair (MVr) devices need preclinical evaluation, which is difficult when compared with valve substitutes because they require MR models for evaluation. This is because the native valve still functions as a valve after the procedure. Previous animal studies have reported the low reproducibility of animal MR models [6–8], limiting the assessment of performance, efficacy and safety of MVr devices. The international standard for cardiac valve repair devices, ISO 5910, outlines *in vitro*, preclinical *in vivo* and clinical evaluations. Although a model that simulates the intended use conditions for MVr devices is recommended, no standard models currently exist. Thus, a reproducible MR model must be developed for research, development and preclinical evaluation of emerging MVr devices.

Various *ex vivo* MR models have been developed. However, most of them only induced small regurgitation, which would not fulfil the interventional indication [9–11]. Moreover, the regurgitation was often uncontrollable and lacked reproducibility. This study aimed to develop a controllable and repairable model of both functional and degenerative MR that fulfils the interventional indication.

## MATERIALS AND METHODS

### Ethical statement

Ethics approval was not required for this study because this was an *ex vivo* (*in vitro*) study.

### Mitral valve models

**Figure 1:**
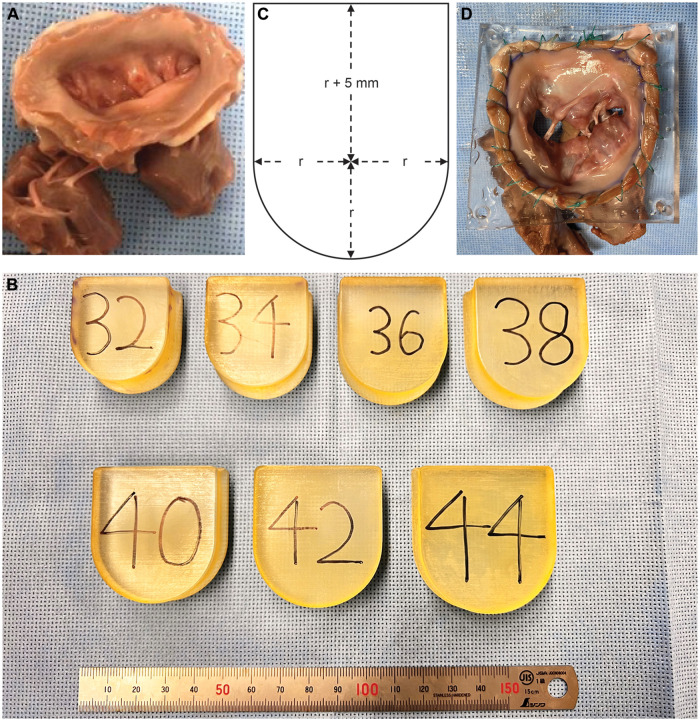
Excised mitral complex and dilators. (**A**) Excised mitral complex including the left atrium, mitral annulus, mitral leaflet, chordae and papillary muscles. (**B**) Original 3D-printed dilators with size variations. (**C**) Geometry data of the dilator. (**D**) Excised mitral complex sutured to the original silicone sheet.

Twenty-five frozen porcine heart samples were obtained from a local abattoir. The mitral complex, which included the left atrium, mitral valve, chordae and papillary muscle, was excised (Fig. 1A). We sought to develop functional mitral regurgitation (FMR) and degenerative mitral regurgitation (DMR) models in separate procedures. Atrial FMR rather than ventricular FMR was mimicked by annular dilatation, whereas DMR was mimicked by cutting the chorda. Each MR model underwent valve repair. We chose the edge-to-edge technique for FMR and artificial chorda implantation for DMR repair, which served as the basis of MitraClip and NeoChord (NeoChord, Inc., St. Louis Park, MN, USA).

### Functional mitral regurgitation *model preparation*

The annulus of the valves was dilated using an original dilator (Fig. 1B and C). Their sizes vary, and their width differed by 2 mm between each size. The dilation procedure was divided into three sets, which were 10 min each. The first dilator was chosen according to the baseline size of the annulus, whereas dilators 1 size larger than the previous set were chosen for the subsequent sets. Throughout the dilation procedure, the annulus was immersed in 0.1% collagenase solution to degenerate the mitral annulus for efficient dilatation. To maintain collagenase activity, the whole dilatation procedure was performed in the incubator kept at 37°C. The annular size was measured again to confirm annular dilatation after the procedure. The valve was then sutured into a silicone sheet (Fig. 1D). The size-selection protocol of the silicone sheet differed by the target MR severity. The sheet chosen was 7-size up and 8-size up compared with the original annulus for the moderate–severe (*n* = 9) and severe (*n* = 6) FMR models, respectively. The FMR models were then repaired with the edge-to-edge technique (5–0, single stitch). The suture was placed where the largest coaptation gap was observed. Half of the severe FMR model received double stitches. Echocardiography was performed on 3 of the 9 moderate–severe FMR models using a cardiology ultrasound system (EPIQ CVx 3D with a Transthoracic Probe; X5-1, Philips, Amsterdam, Netherlands) both before and after the procedures.

To evaluate the effect of collagenase, one of the 25 valves was subjected to histological examination. The valve was sagittally cut into 2 pieces: one without any preparation (control group) and the other immersed in 0.1% collagenase solution for 30 min (collagenase group). The valve was stained with haematoxylin and eosin, Elastica van Gieson and Sirius red.

### Degenerative mitral regurgitation *model preparation*

The valves (*n* = 9) were initially sutured to the flexible silicone sheet. A 4-size up sheet was chosen according to the baseline size of the annulus to mimic annular dilatation in the clinical setting. The size was chosen because it was the largest that did not induce FMR and the smallest that prevented excessive coaptation of the leaflets. Because the valves could be sutured to the sheet without causing any fractures, no treatment of the annulus with the collagenase solution was conducted for this group. After papillary muscle fixation, 6 of the 9 valves’ chorda (P2), which originated in the anterior papillary muscle, was severed. If the regurgitation was insufficient, the chorda from the posterior papillary muscle was also severed. Then, the valve was repaired using the artificial chordae (5–0, single pair). The artificial chordae were sutured to the papillary muscle and leaflet from where the severed chorda originated. The remaining 3 were sent for echocardiographic assessment.

### Model evaluation

After preparation, the valves were incorporated into the mitral position in the pulsatile circulation simulator. Haemodynamic parameters, including aortic pressure, left ventricular pressure, forward flow and regurgitant volume, were measured before and after the valve repair procedure. In accordance with the interventional indication of MR, the models were intended to produce regurgitation equivalent to moderate–severe (regurgitant fraction >40%) and severe (regurgitant fraction >50%) FMR and DMR models, respectively. The coaptation length (A2/P2), the antero-posterior distance and the inter-commissural distance were evaluated by echocardiography.

### Pulsatile flow and pressure circulation system

**Figure 2:**
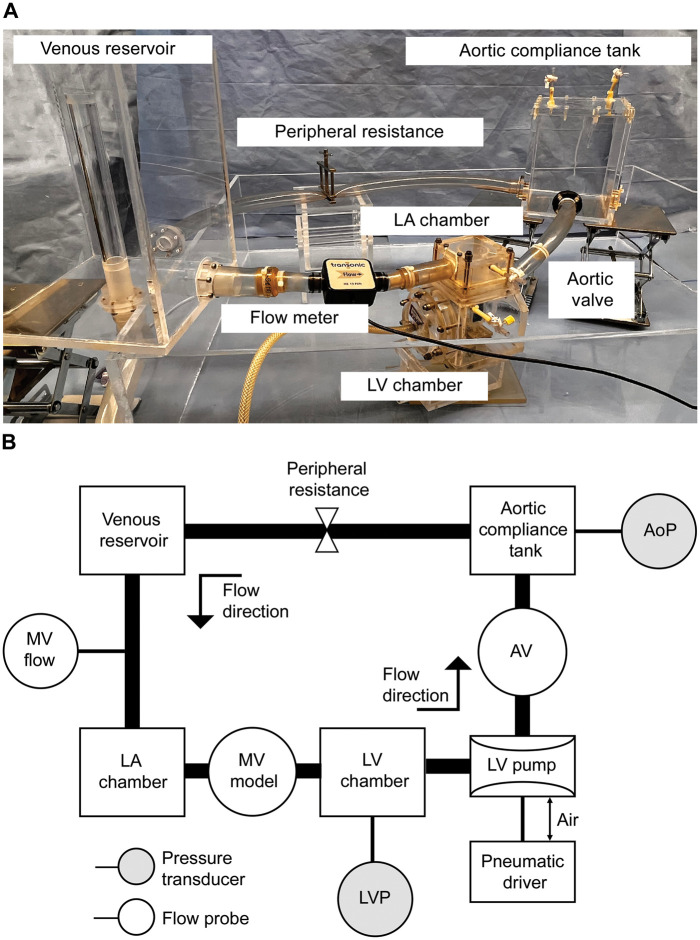
Pulsatile flow and pressure circulation system. (**A**) Picture of the system and (**B**) diagram of the system. AoP: aortic pressure; AV: aortic valve; LA: left atrium; LV: left ventricle; LVP: left ventricular pressure; MV: mitral valve.

The pulsatile flow and pressure circulation system is shown in Fig. 2. Left ventricle (LV) model incorporates a polyurethane diaphragm driven by positive and negative air pressures. A prepared mitral valve was inserted between the left atrium and the LV chambers, whereas the papillary muscles were fixed to the stage inside the LV chamber. The pulse rate was set at 70 beats/min with a systolic fraction of 35%. The haemodynamic target was set to an aortic pressure of 120/80 mmHg and a forward flow of 4.0 l/min. The flow was measured using an ultrasonic flow sensor (ME-PXN ME19PXN325; Transonic, Ithaca, NY, USA) inserted before the mitral valve. Left ventricular and aortic pressures were measured using pressure transducers (PXMK10200; Edwards Lifesciences, Irvine, CA, USA). Data were calculated as averages from 6 consecutive cycles.

### Statistics

The normality of the continuous variables was tested using the Shapiro–Wilk test. Continuous variables are expressed as means and standard deviation (SD) for normally distributed data or medians and interquartile range (IQR) when not normally distributed. A paired *t*-test for normal and a Wilcoxon signed-rank test for non-normal variables were used to evaluate the difference in each parameter before and after the valve preparation and valve repair. Differences were considered significant if the *P*-value was < 0.01. All statistical analyses were performed using the SPSS 18.0 software (SPSS Inc., Chicago, IL, USA). To minimize the bias, a pathological evaluation was performed by 2 specialists independently.

## RESULTS

### *Moderate–severe* functional mitral regurgitation *model*

**Figure 3:**
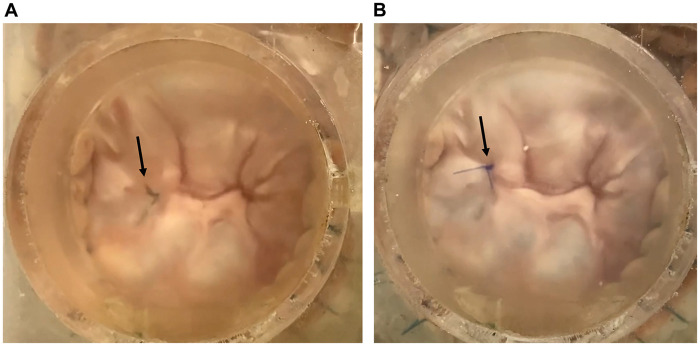
Functional mitral regurgitation model (**A**) before and (**B**) after the valve repair procedure. (**A**) The arrow shows the coaptation gap between the leaflets caused by annular dilatation. (**B**) The arrow shows the edge-to-edge suture technique, which enables coaptation of the leaflets.

The mean annular circumference of the 6 valves was 135.0 (SD: 6.8) mm before the preparation, which was dilated to 148.6 (SD: 7.3) mm after the preparation (*P* < 0.001). A coaptation gap caused by annular dilation was observed in the anterior commissure (Fig. 3A and Video 1), which was successfully repaired by the edge-to-edge technique (Fig. 3B and Video 1).

**Figure 4:**
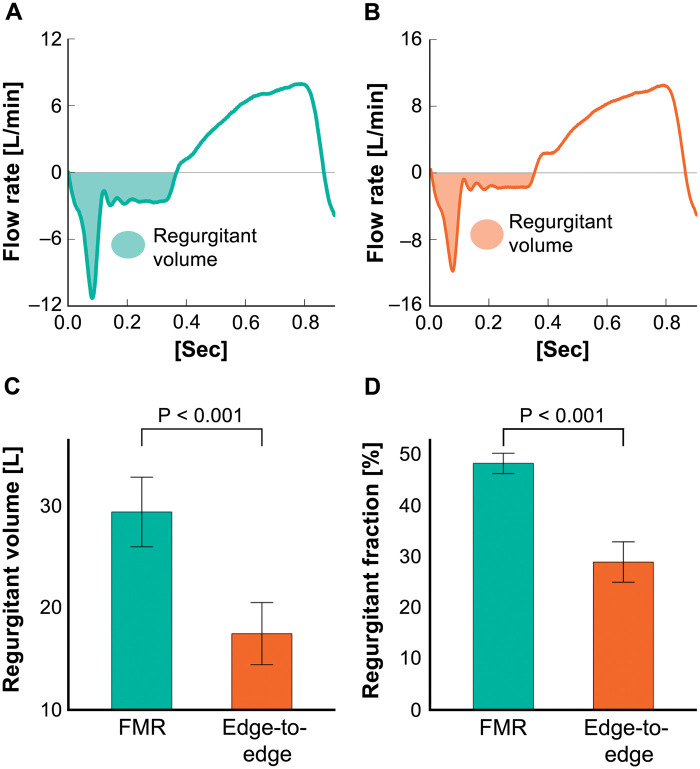
Haemodynamic data of the FMR model before and after valve repair (edge-to-edge technique). (**A**) Representative flow waveform and regurgitant volume before valve repair. (**B**) Representative flow waveform and regurgitant volume after valve repair. (**C**) Mean regurgitant volume before and after valve repair. (**D**) Mean regurgitant fraction before and after valve repair. The error bars show standard error of the mean. FMR: functional mitral regurgitation.

The regurgitant volume and regurgitant fraction were significantly decreased after the edge-to-edge technique [regurgitant volume, 29.2 (SD: 2.8) mL vs 17.4 (SD: 3.4) mL, *P* < 0.001; regurgitant fraction, 47.9% (SD: 2.2%) vs 28.7% (SD: 4.4%), *P* < 0.001] (Fig. 4). The regurgitant fractions before and after the valve repair procedure were equivalent to moderate–severe MR and mild MR in the clinical setting. No difference was observed in aortic pressure and forward flow before and after the valve repair procedure [before versus after: systolic aortic pressure, 120.6 (SD: 1.4) vs 120.2 (SD: 2.7) mmHg, *P* = 0.68; diastolic aortic pressure, 80.4 (SD: 1.7) vs 78.4 (SD: 2.0) mmHg, *P* = 0.20; forward flow, 4.3 (SD: 0.4) vs 4.2 (SD: 0.4) l/min; *P* = 0.42].

### *Severe* functional mitral regurgitation *model*

The median annular circumference of the 6 valves was 142.1 (IQR: 138.5–145.7) mm before the preparation, which dilated to 156.4 (IQR: 152.8–159.9) mm after the preparation (*P* = 0.014). The median systolic pressure, mean diastolic aortic pressure and mean forward flow were 118.6 (IQR: 117.8–119.9) mmHg, 79.9 (SD: 2.4) mmHg and 4.0 (SD: 0.0) l/min, respectively. The mean regurgitant fraction was 59.4% (SD: 6.0%), which was equivalent to severe MR in the clinical setting. Half being repaired with 1 stitch, and the other half with 2 stitches of edge-to-edge suture, mean regurgitant fraction for each improved from 61.2% (SD: 4.0%) to 42.1% (SD: 1.6%) and from 57.6% (SD: 7.0%) to 33.3% (SD: 4.7%).

### Histological analysis

**Figure 5:**
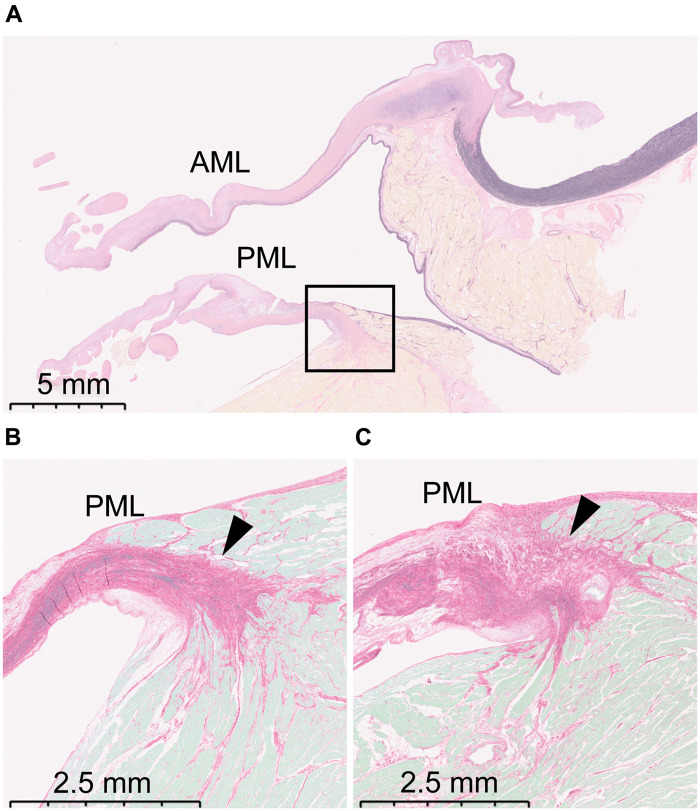
Histology of the valve. (**A**) Overview of the histological section of the control group. The whole section including both the AML and PML is shown. A box surrounding the mitral annulus is inserted, which is the focus of (**B**) and (**C**). Elastica van Gieson staining with original magnification of ×40. The scale bar indicates 5 mm. (**B**) The arrowhead shows the posterior mitral annulus of the control group. Collagen fibres are well-organized. Sirius red staining, with original magnification of ×40. The scale bar indicates 2.5 mm. (**C**) The arrowhead shows the posterior mitral annulus after preparation with the 0.1% collagenase solution. Collagen fibres are fragmented and disorganized, indicating the effect of the collagenase treatment on the tissue. Sirius red staining with original magnification of ×40. The scale bar indicates 2.5 mm. AML: anterior mitral leaflet; PML: posterior mitral leaflet.

The collagen fibre bundle of the posterior annulus was fragmented and disorganized in the collagenase group, which was not changed in the anterior annulus (Fig. 5).

### Degenerative mitral regurgitation *model*

**Figure 6:**
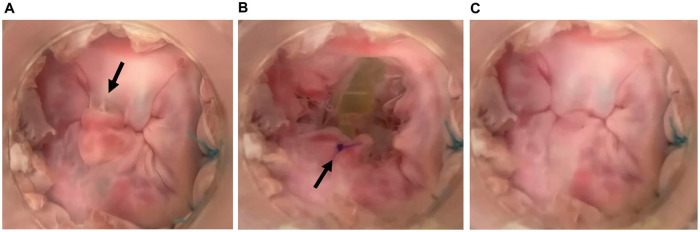
Degenerative mitral regurgitation model. (**A**) The arrow shows the prolapsed P2 segment and torn chordae. (**B**) Diastolic phase of the model after repair with an artificial chorda implanted in the P2 segment. The arrow shows the implanted artificial chorda. (**C**) Systolic phase of the model, showing good coaptation of the repaired P2 segment.

**Figure 7:**
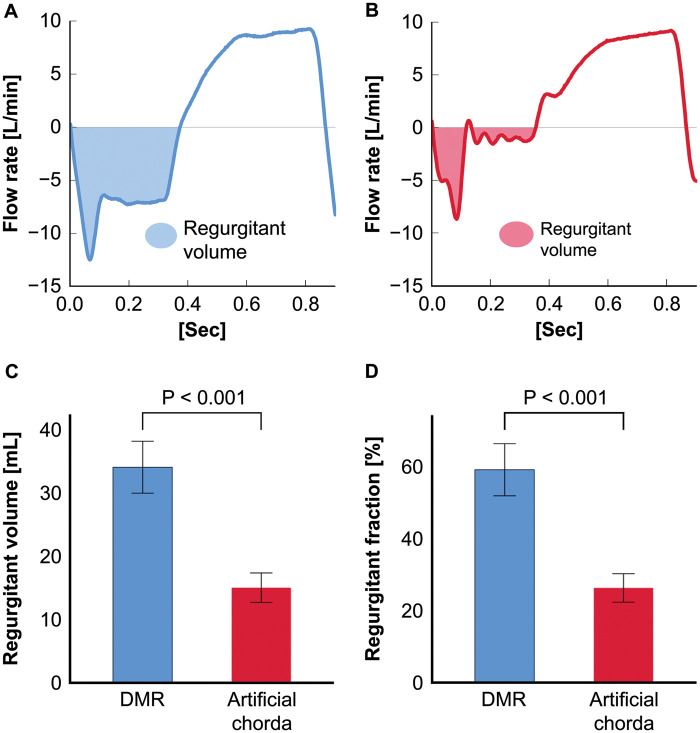
Haemodynamic data of the DMR model before and after valve repair (artificial chorda implantation). (**A**) Representative flow waveform and regurgitant volume before valve repair. (**B**) Representative flow waveform and regurgitant volume after valve repair. (**C**) Mean regurgitant volume before and after valve repair. (**D**) Mean regurgitant fraction before and after valve repair. The error bars show standard error of the mean. DMR: degenerative mitral regurgitation.

The mean baseline annular circumference of the 6 valves before preparation was 133.8 (SD: 6.7) mm. Annular size of the DMR models was not measured because they were sutured to the silicone sheet immediately after the baseline size measurement. Half of the model required additional severance of the chorda originates in the posterior papillary muscle. The prolapsing leaflet (P2) (Fig. 6A and Video 2) was repaired by implanting the artificial chorda (Fig. 6B and C and Video 2). The regurgitant volume and regurgitant fraction significantly decreased after the artificial chorda implantation [regurgitant volume, 33.8 (SD: 4.6) vs 14.9 (SD: 2.6) mL, *P* < 0.001; regurgitant fraction, 58.5% (SD: 8.0%) vs 26.0% (SD: 4.4%), *P* < 0.001] (Fig. 7). Regurgitant fractions were equivalent to severe and mild MR in the clinical setting. No difference was observed in the aortic pressure and forward flow before and after the valve repair procedure [before versus after: systolic aortic pressure, 120.6 (SD: 3.0) vs 119.9 (SD: 2.2) mmHg, *P* = 0.60; diastolic aortic pressure, 82.3 (IQR: 79.8–82.9) vs 81.2 (IQR: 79.0–82.1) mmHg, *P* = 0.046; forward flow, 4.1 (SD: 0.0) vs 4.0 (SD: 0.1) l/min; *P* = 0.060].

### Echocardiographic assessment

The coaptation length (A2/P2) tended to be longer in the DMR model and was increased after valve repair in the FMR model (Table 1). Both the antero-posterior distance and the inter-commissural distance were longer in the FMR model. While the antero-posterior distance decreased after FMR repair, the inter-commissural distance remained unchanged.

**Table 1:** Echocardiographic findings and regurgitant fraction of the models

	DMR before chordal cut	FMR	Repaired FMR
CL (mm)	11.3 (SD: 1.1)	6.1 (SD: 1.2)	7.3 (SD: 1.1)
AP (mm)	20.3 (SD: 0.7)	25.4 (SD: 0.9)	24.0 (SD: 1.0)
IC (mm)	24.9 (SD: 1.1)	27.5 (SD: 0.8)	27.3 (SD: 1.0)
RF (%)	24.0 (SD: 2.2)	49.7 (SD: 4.5)	34.0 (SD: 2.9)

AP: antero-posterior distance; CL: coaptation length; DMR: degenerative mitral regurgitation; FMR: functional mitral regurgitation; IC: inter-commissural distance; RF: regurgitant fraction; SD: standard deviation.

## DISCUSSION

We successfully developed controllable and repairable models of FMR and DMR. The amount of regurgitation was controllable in both models. In the FMR model, the size of the dilator and silicone sheet to which the valve was sutured to was the parameters that can control the regurgitation. The number of resected chordae was a parameter in the DMR model. Both models developed sufficient regurgitant fraction, which fulfilled the indication of the intervention. The regurgitation was significantly reduced by the valve repair procedures, which reflects the feasibility of evaluating valve repair devices. Echocardiographic findings supported the validity of the results.

Previous *ex vivo* models of FMR are classified into (i) models using the whole heart or the left side of the heart [9, 12–16] and (ii) models using only the excised mitral complex [10, 11, 17–20]. In the former type, the anatomical structure of the whole heart is preserved, which enables accurate simulation of the complex physiological characteristics of FMR. However, the use of the whole heart limits the reproducibility of the model. As the annulus and LV interact, it is difficult to quantitatively dilate each component and control MR severity. Moreover, achieving sufficient dilation of healthy heart samples, especially hypertrophic swine hearts, is often difficult when used as a whole heart with all the supporting tissues remained [15]. Several previous studies have reported FMR production [9, 12, 14–16]; however, none of them produced sufficient MR that fulfils the interventional indication. Jaworek *et al.* [13] produced an FMR model by dilating the annulus and LV by pressurizing the LV with a continuous flow pump after clamping the aorta. They used deer hearts instead of swine hearts and achieved a decrease of 53% (1.9 (SD: 0.8) vs 0.9 (SD: 0.9) l/min) in aortic flow without increasing aortic regurgitation in the FMR model compared with the control. However, the aortic flow was distinctly small compared with that of the clinical situation. Moreover, the controllability of the amount of MR is uncertain because the flow data vary widely in the FMR group.

On the contrary, Imbrie-Moore *et al.* [18] produced an iris-like dilatation device and enabled quantitative dilatation of the annulus of the excised mitral complex. They achieved MR with a regurgitant fraction of 47.15% (SD: 7.8%). However, this value was achieved when the annulus was dilated at maximum, which meant that this amount was the upper limit for the model. Another study on an excised mitral complex using a different dilator reported a regurgitant fraction of 23.2% [11].

In the present study, MR severity was controlled, and both moderate–severe and severe FMR with regurgitant fractions of 47.9% (SD: 2.2%) and 59.4% (SD: 6.0%), respectively, were successfully developed. Furthermore, our MR models can be repaired using the edge-to-edge technique for FMR and the artificial chorda technique for DMR models. Given that the current models produce regurgitation meeting the interventional indications for both FMR and DMR, they enable clinicians and engineers to accurately evaluate the performance of their procedures and devices.

An advantage of our protocol is the use of collagenase. Collagenase is an enzyme that breaks the peptide bonds in collagen. As shown in Fig. 5, the collagen fibre bundle around the posterior annulus was rarefied and fractured after immersing in collagenase, which enabled sufficient dilatation of the posterior annulus. A study reported a similar approach using phenol injection to the annulus [12]. It achieved a mitral valve leakage of 0.18 (SD: 0.09) l/min by pressurizing the LV with saline solution injection under a hydrostatic LV pressure of 90 mmHg. However, the leakage volume appeared insufficient to produce a clinically relevant regurgitant fraction for FMR.

Another advantage is the size-independent availability of the native valve. Previous studies often require initial qualification based on the valve size to improve MR control [10, 11, 17–20]. In the present study, instead of restricting the native valve size, the dilator and silicone sheet were adjusted based on the size of the native valve. This increases the efficiency of MR model preparation.

If MR models can be successfully produced in *in vivo* animal models, they may be the best for preclinical studies. However, previous studies have indicated difficulty in producing *in vivo* models of MR [6–8, 21–25]. Although Hill *et al.* [21] had successfully produced severe DMR in all 12 sheep by cutting the P2 chordae under direct vision, none of them could be weaned off the cardiopulmonary bypass and required immediate MR correction. In this model, the optimal evaluation of transcatheter devices, which is usually performed under spontaneous circulation, is difficult. Other studies using swine [22, 23] have reported chordae severance under spontaneous circulation guided by real-time echocardiography or fluoroscopy. Real-time guidance of MR under spontaneous circulation allowed appropriate severance of the chordae. However, 10 of 26 (22) and 3 of 11 (23) swine died before study completion. The development of *in vivo* FMR models tended to be more difficult. In most studies, iatrogenic myocardial ischaemia models have been made to produce FMR [6, 7, 24, 25]. Unlike DMR models, FMR developed ∼7–8 weeks after the procedure. Because the lesion involves not only the valve but also the LV and that MR development takes time, *in vivo* FMR models exhibited lower productivity, only developing 6, 7 and 10 feasible FMR models producing MR > moderate out of 31, 12 and 24 subjects, respectively [6, 7, 25]. Most of the excluded samples died before study completion. Unlike these studies, Yamauchi *et al.* [8] developed FMR by making multiple incisions to the mitral annulus. Without making myocardial ischaemia, they succeeded in keeping all 7 subjects alive; however, MR > moderate developed in only 2 of the 7 subjects. Thus, *in vivo* MR models are time-consuming and difficult to make, requiring many samples leading to high costs. Therefore, the appropriate use of *in vitro* (including *ex vivo*) and *in vivo* models in accordance with study purposes is imperative to evaluate the performance, efficacy and safety of the valve repair procedures and devices.

### Limitations

This study had several limitations. One limitation of our study is its *ex vivo* nature, which limits the incorporation of real-world surgical scenarios for mitral valve repair. However, our primary objective in the development of the MR models was the evaluation of novel techniques and devices. In this context, we believe the model offers a valuable contribution. For the DMR model, we specifically focused on P2 prolapse because it is the most common segment involved in DMR. We have developed a model for non-A2P2 prolapse. Assessments of a novel device idea are currently being conducted using the model. For the FMR model, we simulated symmetric annular dilatation, where ischaemic MR is commonly associated with asymmetric dilatation. However, asymmetric dilatation can be simulated in the same protocol using an asymmetric dilator and silicone sheet, which we are planning to carry out in the future. Another limitation of the FMR model is that we focused only on the annulus. As FMR is typically associated with ventricular dilatation leading to papillary muscle displacement and chordal tethering, simulation of these changes in the LV may allow a more accurate disease simulation. Moreover, the anchoring and sealing site of TMVr devices cannot be evaluated in the present model because it does not contain the whole atrium and LV. Nevertheless, our model can evaluate the efficacy of most of the surgical MVr procedures such as the artificial chordae technique, procedures on the leaflet including the edge-to-edge technique and ring annuloplasty (which is not presented in the present study). Most of the existing TMVr devices are based on these conventional surgical procedures; therefore, proof-of-concept studies of novel TMVr devices could be done on this model. Further studies on the development of a circulation system, capable of measuring the trans-mitral gradient and the chordal force, may be important for evaluating MVr devices. The use of our MR models that meet the interventional indications may provide valuable information on the efficacy of devices designed to reduce MR.

## CONCLUSIONS

We successfully developed *ex vivo* models of both FMR and DMR that meet the interventional indication. These models proved repairable, which allow for the preclinical evaluation of valve repair procedures and devices. Furthermore, excellent controllability and reproducibility of MR severity could help overcome the challenges associated with *in vivo* animal models. The appropriate use of our *ex vivo* model and *in vivo* animal models may contribute to the reduction of the number of *in vivo* animal experiments, accelerating the developmental speed of novel devices and ultimately benefiting untreated patients with MR.

## Data Availability

The data underlying this article will be shared on reasonable request to the corresponding author.

## ABBREVIATIONS

DMR: Degenerative mitral regurgitation
FMR: Functional mitral regurgitation
IQR: Interquartile range
LV: Left ventricle
MR: Mitral regurgitation
MVr: Mitral valve repair
SD: Standard deviation
TMVr: Transcatheter mitral valve repair
